# A boy with developmental delay and mosaic supernumerary inv dup(5)(p15.33p15.1) leading to distal 5p tetrasomy – case report and review of the literature

**DOI:** 10.1186/s13039-018-0377-1

**Published:** 2018-05-09

**Authors:** Pavel Tesner, Jana Drabova, Miroslav Stolfa, Martin Kudr, Martin Kyncl, Veronika Moslerova, Drahuse Novotna, Radka Kremlikova Pourova, Eduard Kocarek, Tereza Rasplickova, Zdenek Sedlacek, Marketa Vlckova

**Affiliations:** 1 Department of Biology and Medical Genetics, Charles University 2nd Faculty of Medicine and University Hospital Motol, V Uvalu 84, 15006 Prague 5, Czech Republic; 2 Department of Paediatric Neurology, Charles University 2nd Faculty of Medicine and University Hospital Motol, V Uvalu 84, 15006 Prague 5, Czech Republic; 3 Department of Radiology, Charles University 2nd Faculty of Medicine and University Hospital Motol, V Uvalu 84, 15006 Prague 5, Czech Republic

**Keywords:** 5p tetrasomy, Marker chromosome, Mosaicism, Intellectual disability

## Abstract

**Background:**

With only 11 patients reported, 5p tetrasomy belongs to rare postnatal findings. Most cases are due to small supernumerary marker chromosomes (sSMCs) or isochromosomes. The patients share common but unspecific symptoms such as developmental delay, seizures, ventriculomegaly, hypotonia, and fifth finger clinodactyly. Simple interstitial duplications leading to trisomies of parts of 5p are much more frequent and better described. Duplications encompassing 5p13.2 cause a defined syndrome with macrocephaly, distinct facial phenotype, heart defects, talipes equinovarus, feeding difficulties, respiratory distress and anomalies of the central nervous system, developmental delay and hypotonia.

**Case presentation:**

We present a boy with dysmorphic features, developmental delay, intellectual disability and congenital anomalies, and a mosaic sSMC inv dup(5)(p15.33p15.1). He is the fourth and the oldest reported patient with distal 5p tetrasomy. His level of mosaicism was significantly different in lymphocytes (13.2%) and buccal cells (64.7%). The amplification in our patient is smaller than that in the three previously published patients but the only phenotype difference is the absence of seizures in our patient.

**Conclusions:**

Our observations indicate that for the assessment of prognosis, especially with respect to intellectual functioning, the level of mosaicism could be more important than the extent of amplification and the number of extra copies. Evaluation of the phenotypical effect of rare chromosomal aberrations is challenging and each additional case is valuable for refinement of the genotype-phenotype correlation. Moreover, our patient demonstrates that if the phenotype is severe and if the level of sSMC mosaicism is low in lymphocytes, other tissues should be tested.

**Electronic supplementary material:**

The online version of this article (10.1186/s13039-018-0377-1) contains supplementary material, which is available to authorized users.

## Background

Tetrasomies of a part or the whole 5p belong to rare postnatal findings and can be the result of a small supernumerary marker chromosome (sSMC) with inverted duplication (inv dup) or an isochromosome. Clinical outcomes of sSMCs vary from an unaffected to a severely affected status with major anomalies and intellectual disability (ID). sSMCs are often present in mosaics and can even be absent in some tissues. The clinical manifestation is frequently influenced by the level of mosaicism in specific tissues [1].

Only 11 postnatal patients of 5p tetrasomy have been reported, seven with supernumerary i(5)(p10) [2–8], three with amplification of the distal part of 5p – one interstitial triplication [9] and two supernumerary inv dup [10, 11], and one patient had proximal 5p tetrasomy but his phenotype description and sSMC characterization were rather incomplete [12]. The features of 5p tetrasomy are developmental delay, seizures, ventriculomegaly, hypotonia, short stature or growth delay, and fifth finger clinodactyly [11]. A single transverse palmar crease, recurrent infections, abnormalities of the diaphragm, abnormalities of the pinna, microretrognathia, abnormalities of the philtrum and thin upper lip vermilion are also frequently observed. Interestingly, the phenotype seems to be rather similar irrespective of whether the whole 5p or just its distal part is amplified.

In contrast to 5p tetrasomies, partial or complete 5p trisomies are much more frequent. Proximal 5p trisomies are associated with a specific phenotype with macrocephaly, facial anomalies (upslanted palpebral fissures, hypertelorism, epicanthus, depressed nasal bridge, midface retrusion, micrognathia, and abnormality of the pinna), short neck, hypoplasia of the abdominal wall musculature, congenital heart defects, talipes equinovarus, feeding difficulties, respiratory distress, recurrent respiratory infections, and anomalies of the central nervous system (CNS), especially ventriculomegaly. Generalized hypotonia, seizures and ID are also common [13]. The critical region for this phenotype was primarily placed to 5p11-p13 by Chia et al. [14] and later refined to 5p13.1-p13.3 by Loscalzo et al. [15]. The 5p13.2 duplication syndrome was finally defined by Yan et al. [16], and *NIPBL* was suggested as its critical gene [17].

We present a 5-year-old boy with dysmorphic features, feeding difficulties, hypotonia, developmental delay, ID, autistic features, and mosaic sSMC inv dup(5)(p15.33p15.1) resulting in tetrasomy of distal 5p not involving the 5p13.2 region. We compared the genotype and phenotype of our patient with previously described patients of 5p tetrasomy and reviewed the literature for clinical effects of tetrasomy and trisomy of distinct parts of 5p. We found just slight and non-specific differences among the phenotypes of the patients compared. Our observations indicate that for the assessment of prognosis, especially with respect to intellectual functioning, the level of mosaicism could be more important than the extent of the region amplified and the number of extra copies.

## Case presentation

### Clinical report

The boy was born via spontaneous delivery from the second uneventful pregnancy to healthy unrelated Caucasian parents. The age of the mother and the father was 31 and 32 years, respectively. Birth weight was 3650 g (82nd centile) and birth length was 53 cm (87th centile). No congenital anomalies were observed in the newborn, and the neonatal period was unremarkable. During the infant period the boy suffered from feeding difficulties and recurrent upper airways infections. His psychomotor development was delayed. Pyelectasia, foramen ovale apertum and hiatal hernia were detected at the age of 14 months, and brain MRI revealed a cyst in posterior cranial fossa.

**Fig. 1:**
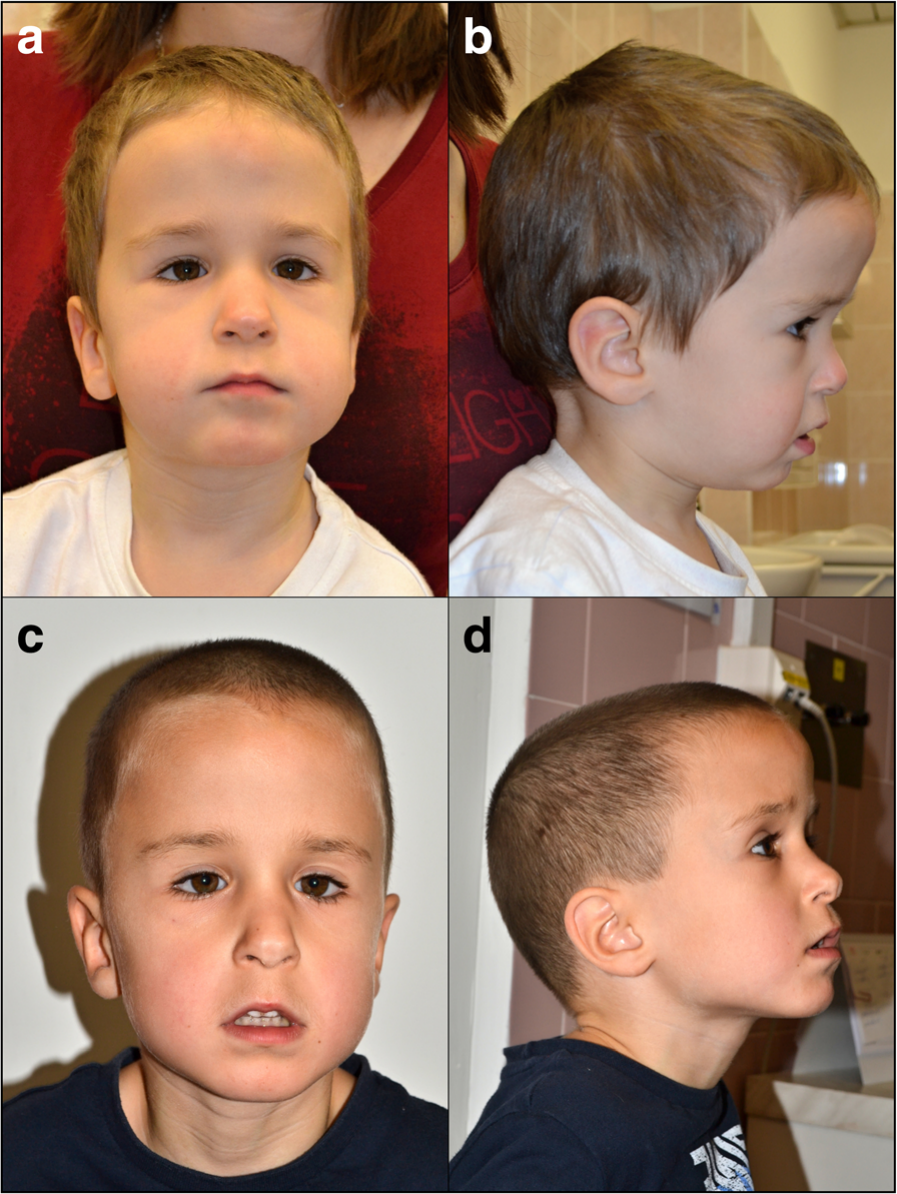
The patient at the age of 3 years and 5 months (**a**, **b**) and 5 years and 1 month (**c**, **d**). Apparent features include low set ears, hypotelorism, downslanted palpebral fissures, epicanthus, depressed nasal bridge, low hanging columella, long philtrum, and thin upper lip vermilion. While short chin is present only at younger age, frontal bossing is more remarkable at older age

He was examined again at the age of 3 years and 5 months due to global developmental delay. He was not able to walk but he started to stand with support. The speech was absent and he used only vocalizations. He had problems with chewing and he could eat only mashed food. His parents described frequent aggressive behavior with biting. Hypotonia (central hypotonic syndrome), hypermobility of joints and pedes planovalgi were also present. His height was 96 cm (11th centile), weight was 13.5 kg (7th centile) and head circumference was 52.5 cm (96th centile). Dysmorphic features included dolichocephaly, frontal bossing, low set ears, abnormality of the pinna, hypotelorism, downslanted palpebral fissures, epicanthus, depressed nasal bridge, low hanging columella, long philtrum, thin upper lip vermilion, short chin, slight midface retrusion, single transverse palmar crease on the right hand, pectus excavatum, and kyphosis (Fig. 1a, b).

At the last examination at the age of 5 years and 1 month his height was 110 cm (18th centile; however, according to growth prediction from the mid parent height, the height of the boy would be expected to be around 97th centile, and thus the actual observation might rather indicate a more significant growth delay), weight was 15.5 kg (2th centile) and head circumference was 54 cm (95th centile). Speech was still absent and feeding and chewing difficulties persisted, but the boy was able to walk independently. He showed symptoms of an autism spectrum disorder such as stereotypical hand movements, frequent bruxism and a very sporadic eye contact. Macrocephaly, dolichocephaly, central hypotonic syndrome and hypermobility of the joints persisted. Pedes planovalgi were accompanied by valgus deformity of the knees. In addition, a small umbilical hernia was present. Midface retrusion and short chin observed previously were not present, but the forehead was even more prominent (Fig. 1c, d).

### Laboratory analysis

The research was prospectively reviewed and approved by a duly constituted ethics committee. Informed consent was obtained from the parents of the patient. Examination of G-banded chromosomes from peripheral blood lymphocytes was performed using standard protocols. For fluorescence in situ hybridization (FISH) analysis of blood lymphocytes and buccal cells, Cytocell Aquarius Cri-du-chat and SOTOS Probe Combination (LPU 013) (Oxford Gene Technology, UK) was used. High-resolution array comparative genomic hybridization (aCGH) and single nucleotide polymorphism (SNP) analysis of the lymphocyte genomic DNA isolated using AutoGen Flex STAR (Autogen, USA) employed the SurePrint G3 ISCA V2 CGH 8x60K Microarray and the SurePrint G3 ISCA CGH + SNP 4x180K Microarray, respectively, according to the protocol of the manufacturer (Agilent Genomics, USA).

### Results

**Fig. 2:**
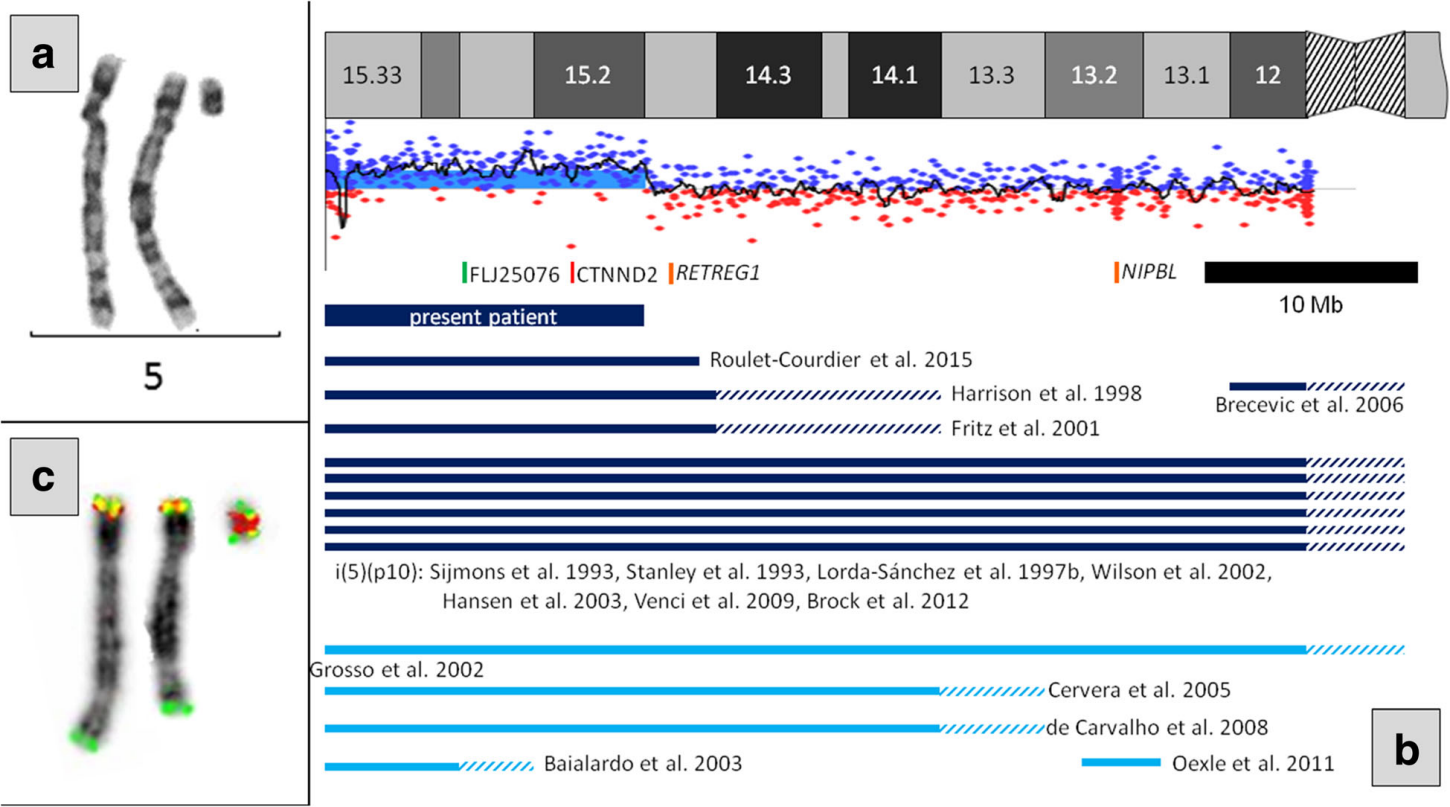
**a** Partial G-banded karyotype of normal chromosomes 5 and the sSMC. **b** Scheme showing the banding pattern of 5p, the result of array CGH in the present patient, FISH probes for the cri-du-chat region employed in our study (5p15.31 (FLJ25076) in green, 5p15.2 (CTNND2) in red), disease-causing genes discussed (orange), the extent of 5p amplification in the present patient (thick dark blue bar) and previously published tetrasomies (inv dups, isochromosomes, an interstitial triplication; thin dark blue bars) and trisomies (terminal and interstitial duplications, unbalanced translocations; thin light blue bars). Uncertain ranges in published patients are hatched. A megabase scale bar is also shown. **c** FISH examination of normal chromosomes 5 and the sSMC. See (**b**) for 5p probe colors. The 5q35 (NSD1) probe is in green. The pattern of signals on the sSMC indicates its structure of inv dup of distal 5p

The patient had a mosaic sSMC and karyotype mos 47,XY,+mar[10]/46,XY[28] (Fig. 2a). Parental karyotypes were normal and the sSMC occurred de novo. aCGH analysis of the patient revealed gain of material in 5p15.33p15.1, in the region chr5:22149_15009591 (hg19) (Fig. 2b). FISH analysis confirmed the origin of the sSMC from 5p15.33p15.1 and revealed its inv dup structure (Fig. 2c), with the final karyotype mos 47,XY,+dup(5)(pter→p15.1::p15.1 → pter)dn[10]/46,XY[28]. The region of tetrasomy was 15 Mb long and encompassed 137 genes including 54 protein-coding genes, of which 14 are known disease-causing genes (Additional file 1). Considering the tetrasomic nature of the aberration, the mean log_2_ratio of 0.176 observed in aCGH indicated a mosaic of 13.0% according to the formula by Valli et al. [18]. FISH analysis of 409 cell nuclei of peripheral lymphocytes showed the sSMC in 13.2% of cells. In contrast, interphase FISH analysis of 312 nuclei of buccal mucosa cells showed the sSMC in 64.7% of cells. SNP array CGH analysis identified 71 at least partly informative SNPs in the interval of the 5p tetrasomy allowing to deduce the parental origin of the sSMC. The analysis was complicated by the low level of mosaicism of the sSMC in lymphocytes, and the almost 1% error rate of the array as indicated by genome-wide discrepancies with Mendelian inheritance. Nevertheless, 18 informative SNPs clearly indicated the paternal origin of the sSMC, and 51 additional SNPs were not at odds with this scenario. Of the SNPs which could discriminate between the paternal alleles, 10 clearly indicated that both SNP alleles on the sSMC were identical to the allele present on the normal chromosome 5 inherited by the patient from the father, and 8 additional SNPs were not at odds with this scenario (showing that the sSMC alleles could originate either from the paternal chromosome 5 inherited by the patient, or from the other paternal chromosome 5). Of the 51 SNPs which were not at odds with the paternal origin, 20 showed alleles identical to the sSMC and the normal paternal chromosome 5, and 29 could not discriminate between the two scenarios.

## Discussion and conclusions

According to literature reports, isochromosomes of the whole 5p (i(5)(p10)) [2–8] are more frequent than inv dups of distal 5p [9–11]. Our patient is the fourth and the oldest case with tetrasomy of distal 5p. He is the only patient old enough to allow proper evaluation of his intellectual functioning, not only developmental delay, but also of all other features.

Phenotypes of the tetrasomic patients are similar and only few unspecific differences can be observed (Additional file 2). Recurrent respiratory infections were observed in our patient and in two other patients with distal 5p tetrasomy [10, 11], and another patient died at the age of 35 days because of bronchopneumonia [9]. Respiratory infections were also described in a patient with mos i(5)(p10) [4]. A possible explanation for this feature was proposed in a patient with trisomy of the whole 5p by Grosso et al. [19], who found low level of secretory immunoglobulins A (IgA). Nevertheless, detailed immunological examination was not performed and the finding could be coincidental. Patients with 5p tetrasomy published in the literature were not tested for IgA and in our patient it was within the normal range. More detailed immunological examination of patients with 5p amplifications is needed to elucidate the cause of recurrent infections.

Compared to the other patients with distal 5p tetrasomy [9–11], the tetrasomy in our patient does not encompass six of their amplified genes. The previous patients manifested seizures in infancy but our patient did not, and thus one of these genes could cause the seizures. Inactivating *RETREG1* mutations cause autosomal recessive hereditary sensory and autonomic neuropathy IIB (OMIM #613115), but our patient does not show this phenotype. No diseases are associated with the other genes. While the *FBXL7* [20]*, MARCH11* [21]*, ZNF622* [22], and *BASP1* [23] genes are expressed in brain and could possibly contribute to seizures, no data exist on *MYO10* expression in CNS.

The comparison of phenotypes associated with 5p tetrasomy and 5p trisomy is summarized in Additional file 3. The common features of all 5p amplifications are anomalies of CNS, hypotonia, seizures, and ID. Although the critical region of the 5p13.2 duplication syndrome is involved neither in the tetrasomic region of our patient nor in the distal 5p tetrasomy regions of the previous patients [9–11], many of their traits are similar to those of patients with the 5p13.2 duplication syndrome, including congenital heart defects and seizures (in two and three of the four distal 5p tetrasomy patients, respectively). The presence of these symptoms in patients with tetrasomies not involving *NIPBL* indicates that other gene(s) for these conditions may exist in 5p14 and 5p15. Phenotypic overlap among carriers of amplifications of non-overlapping regions of 5p was noted by Cervera et al. [24]. Their patient with dup(5)(p13.3p15.3) had severe ID, seizures, macrocephaly, upslanted palpebral fissures, epicanthal folds, depressed nasal bridge, and abnormal pinna, which are also characteristic for the 5p13.2 duplication syndrome. Also de Carvalho et al. [25] reported t(5;15)(p13.3;p12) leading to distal 5p trisomy in five individuals with features resembling the 5p13.2 duplication syndrome. This reopens the question if the 5p trisomy phenotype is caused solely by trisomy of 5p13.2 with *NIPBL* as the possible candidate gene [26]. Conversely, also a normal individual with a terminal 5p trisomy resulting from der(15)t(5;15)(p15.1;p13) was reported [27].

The tetrasomy in our patient affected a total of 137 genes. Extensive data collected on their disease involvement, expression and sensitivity to variation are in Additional file 1. The majority of the protein-coding genes are expressed in all tissues including the brain, and some of them show lack of tolerance to loss-of-function variation. Evidence for sensitivity to increased copy number is generally sparser, and the ClinGen Dosage Sensitivity Map (https://www.ncbi.nlm.nih.gov/projects/dbvar/clingen/) indicates triplosensitivity in none of these genes. Evidence exists for haploinsufficiency of *TERT* and *CTNND2*, but neither the phenotype caused by the loss of these genes nor a “mirror” phenotype could be observed in our patient. The *TRIO* gene has the strongest evidence for haploinsufficiency and causes autosomal dominant mental retardation 44 (MRD44, OMIM #617061). Our patient and also other patients with 5p tetrasomy showed some features of this disorder such as downslanting palpebral fissures, short nose, micrognathia, facial asymmetry, large ears, clinodactyly, feeding difficulties, developmental delay, ID, poor speech, autistic features and recurrent infections, while microcephaly and seizures are present only in some of them [9–11].

Moreover, according to the STRING database (https://string-db.org/), the TRIO protein activates RAC1, and the *RAC1* gene causes autosomal dominant mental retardation 48 (MRD48, OMIM #617751) with a very variable phenotype. Other possibly interesting protein interactions may exist. For example, the *NDUFS6* gene amplified in our patient encodes the NADH:ubiquinone oxidoreductase subunit S6 of the mitochondrial complex I (MCI). Biallelic *NDUFS6* mutations cause MCI deficiency (OMIM #252010) but no evidence exists for the sensitivity of this gene to amplification. A duplication of *NDUFS4*, an interaction partner of *NDUFS6*, in case 331431 from DECIPHER was considered to be possibly pathogenic. The phenotype and severity of MCI deficiency is highly variable and it is difficult to decide if the phenotype of our patient could fit at least partly this condition because of his rather unspecific symptoms; his phenotype is definitely not discordant with mild MCI deficiency. Similarly, the product of *MED10* is a component of the Mediator complex, a coactivator for DNA-binding factors activating transcription by RNA polymerase II [28]. Mutations in a possible interacting partner of *MED10*, *MED12*, cause X-linked ID (OMIM #309520, #300895, #305450). Another highly interconnected protein encoded by the sSMC is CCT5, a subunit of the chaperonin containing TCP1 complex involved in folding of cytoskeletal proteins. Multiple subunits of this complex have been associated with various neurodevelopmental disorders [29, 30]. It must be noted that it is unclear from in silico analysis how much the levels of proteins encoded by genes in the amplified interval are changed, if these changes cause any disturbances of stechiometric ratios in the protein complexes, and if these disturbances impair the functions of these complexes.

As the sSMC does not contain the normal chromosome 5 centromere, it is likely to carry a neocentromere (reviewed in [31]). A variable postzygotic mitotic stability of the sSMC in individual tissues (possibly due to the differences in the time of neocentromere activation [32]) or a different proliferation disadvantage of different cell types with the sSMC could cause the different level of mosaicism observed in lymphocytes and buccal cells of our patient. The SNP array CGH analysis revealed the paternal origin of the sSMC and showed that the two copies of distal 5p present on the sSMC are identical with the corresponding part of the normal paternal chromosome 5 present in the patient. This could point to the paternal meiotic origin of the sSMC from an acentric fragment via hairpin formation, reduplication and neocentromere acquisition described by Murmann et al. [32]. In contrast to usually non-mosaic 5p trisomies, the phenotype of 5p tetrasomy due to a sSMC can be influenced by the level of mosaicism. Three patients with mosaic i(5)(p10) in fibroblasts but a normal karyotype in lymphocytes were reported. A girl had a 62% mosaic of i(5)(p10) in fibroblasts and developmental delay, seizures, mild ID and hypotonia [3]. Another girl with a milder phenotype (borderline intelligence and psychomotor skills, but with complex partial seizures) had only 10% mosaic in fibroblasts [6]. Finally, a boy having in addition ventriculomegaly, short stature, and macrocephaly, had a 14% mosaic in fibroblasts [8]. All three children exhibited mosaic hyperpigmentation of skin, and therefore the biopsy should target the hypo- or hyperpigmented lesions generally following the lines of Blaschko [33]. Skin biopsy can be replaced by buccal smear but if the examination is negative and the suspicion for mosaicism persists, invasive sampling is necessary to reach the diagnosis. Because aCGH cannot distinguish trisomy from 50% mosaic of tetrasomy, FISH should be used to elucidate the type of aberration. However, in contrast to i(5)(p10) cases, skin signs of mosaicism have not been observed in patients with mosaic distal 5p tetrasomy. The pitfalls of mosaicism are illustrated also by an infertile man with no dysmorphic features, ID nor congenital defects who carried mos i(5)(p10) in 16% of lymphocytes, but no such sSMC in skin fibroblasts and urothelial cells [7]. The similarly low level of mosaicism in lymphocytes of our patient initially also shed doubts on its causality, until the buccal analysis was performed which showed a much higher percentage of cells carrying the sSMC. Similarly to Pallister-Killian syndrome with supernumerary isochromosome 12p in skin fibroblasts (and other tissues), but with no or insignificant representation in lymphocytes [34], our case showed that the examination of another tissue is essential, and the easily accessible buccal cells could serve this aim. The decision if the sSMC is causal or if it is just a coincidental finding not explaining the phenotype is very important as it is crucial for planning of possible additional examinations.

## Additional files

Additional file 1: **Table S1.** Detailed description of encompassed genes. (XLS 328 kb)

Additional file 2: **Table S2.** Comparison of clinical features of patients with 5p tetrasomies. (XLS 41 kb)

Additional file 3: **Table S3.** Comparison of phenotypes caused by distal 5p tetrasomy, i(5)(p10) and dup5p13.2. (XLS 42 kb)

## Abbreviations

aCGH: Array comparative genomic hybridization
CNS: Central nervous system
FISH: Fluorescence in situ hybridization
inv dup: Inverted duplication
SNP: Single nucleotide polymorphism
sSMC: Small supernumerary marker chromosome
